# Noisy neighbourhoods: quorum sensing in fungal–polymicrobial infections

**DOI:** 10.1111/cmi.12490

**Published:** 2015-09-03

**Authors:** Emily F. Dixon, Rebecca A. Hall

**Affiliations:** ^1^ Institute of Microbiology and Infectionand School of Biosciences, University of BirminghamEdgbastonBirminghamB15 2TTUK

## Abstract

Quorum sensing was once considered a way in which a species was able to sense its cell density and regulate gene expression accordingly. However, it is now becoming apparent that multiple microbes can sense particular quorum‐sensing molecules, enabling them to sense and respond to other microbes in their neighbourhood. Such interactions are significant within the context of polymicrobial disease, in which the competition or cooperation of microbes can alter disease progression. Fungi comprise a small but important component of the human microbiome and are in constant contact with bacteria and viruses. The discovery of quorum‐sensing pathways in fungi has led to the characterization of a number of interkingdom quorum‐sensing interactions. Here, we review the recent developments in quorum sensing in medically important fungi, and the implications these interactions have on the host's innate immune response.

## Introduction

Fungi and bacteria often occupy the same niche, whether in the environment, or in plant or animal hosts. The evolution of eukaryotes, both fungi and mammalian hosts, has therefore been heavily influenced by the close proximity of bacteria. Interactions between bacteria and fungi can be chemical, for example, quorum sensing (QS), a cell–cell communication mechanism, or physical, including coaggregation within a biofilm. Polymicrobial interactions are of great importance in a variety of fields. For example, in the food industry, interactions between lactic acid‐producing bacteria and yeasts are important in the production of baked goods (Gobbetti, 1998). In the dairy industry, interactions between yeasts and bacteria are important factors in fermented products and in the ripening of specific cheeses (Viljoen, 2001). In agriculture, polymicrobial interactions play an important role in the complex mycorrhizal network of economically important crops and plants (Deslandes *et al*., 2003; Bonfante and Anca, 2009; Newton *et al*., 2010). Finally, polymicrobial interactions have important consequences in veterinary and human medicine (Peleg *et al*., 2010).

Clinically, polymicrobial infections are harder to treat because of increased resistance to antimicrobial therapy, and as such, polymicrobial diseases can have increased mortality compared with their monomicrobial counterparts (McKenzie, 2006; Harriott and Noverr, 2011). For example, a recent review of polymicrobial bloodstream infections (BSIs) within an intensive care unit found that polymicrobial BSIs had a mortality rate of 47% compared with 19.6% of monomicrobial BSIs (Pammi *et al*., 2014). Despite this, we currently have limited understanding of the roles of these interactions in disease progression. Therefore, characterizing the complex interactions that occur in these mixed species communities is essential to provide alternative therapies for the treatment of individuals with polymicrobial disease.

Fungal–bacterial interactions vary in dynamics depending on species, strain and environment, but they can be endosymbiotic, synergistic or antagonistic. For example, the plant fungal pathogen *Rhizopus microsporus* has an endosymbiotic relationship with the Gram‐negative bacterium *Burkholderia rhizoxinica* and *Burkholderia endofungorum*, using the bacteria to produce rhizoxin, the cause of rice seedling blight (Partida‐Martinez and Hertweck, 2005; Partida‐Martinez *et al*., 2007). Interactions during dental plaque formation tend to be synergistic, promoting biofilm formation (Diaz *et al*., 2012; Nobbs and Jenkinson, 2015). *Penicillium* species are known to produce quorum‐sensing inhibitors to prevent bacterial communication, reducing their competitors virulence (Rasmussen *et al*., 2005).

While we acknowledge that the gut is a major site for interkingdom interactions, which are essential in maintaining homeostasis (Hooper and Gordon, 2001; Fujiya *et al*., 2007) and regulating immunity (Kau *et al*., 2011), this review will focus on the role of fungal QS in medically important polymicrobial infections and discuss the impact of these microbial signalling molecules on the host's immune system.

## Polymicrobial infections involving fungi

**Figure 1:**
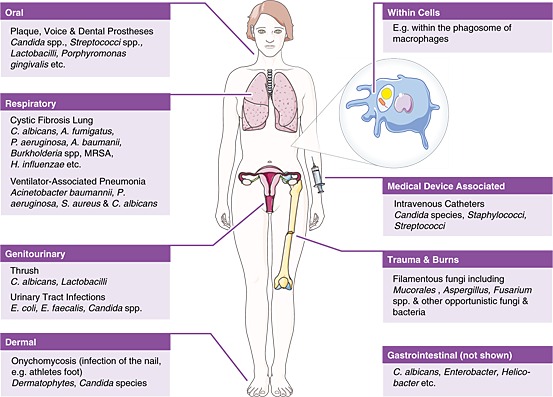
Common niches in which fungal quorum‐sensing interactions occur. Diagrammatic representation of the most common niches where polymicrobial interactions occur. Only key species are highlighted.

Fungal–polymicrobial interactions are important in a variety of disease states and niches including, but not limited to, infections of the respiratory system [i.e. cystic fibrosis (CF) and ventilated‐associated pneumonia], formation of dental plaque, invasive disease, skin and mucosal infections, and bloodstream infections (Fig. 1) (reviewed in Peleg *et al*., 2010; Frey‐Klett *et al*., 2011). For example, the CF lung is a major site for polymicrobial infections. Although *Pseudomonas aeruginosa* is the major colonizer of the CF lung, *Burkholderia cepacia* complex and *Staphylococcus aureus* also predominate in these infections, with colonization of *B*. *cepacia* indicating chronic infection (Jones *et al*., 2004). Fungi also colonize the CF lung with *Candida albicans*, *Aspergillus fumigatus* and *Scedosporium* species being the most frequently observed (Bakare *et al*., 2003; Chmiel *et al*., 2014). Therefore, like the gut, the CF lung is a major site for interkingdom interactions. However, the occurrence of polymicrobial infections in other niches is often under‐reported, because of difficulties in diagnosing multiple pathogens via traditional culture techniques (McKenzie, 2006; Rolston *et al*., 2007; Chotirmall *et al*., 2010). To further compound this issue, fungal disease is also under‐reported, especially when the comparative burden on society is taken into account (Head *et al*., 2013). Advances in next generation sequencing have enabled efficient diagnosis of polymicrobial disease (Harris *et al*., 2007; Sibley *et al*., 2008; Mohammadi *et al*., 2015). Still, many studies focus on 16S ribosomal sequencing, therefore, missing out any fungal or other eukaryotic species that may be present. A combination of sequencing techniques must be used to better understand the full range of species present in a given disease.

## Quorum sensing

Microbes were once thought to act selfishly, but it is now accepted that populations can function cooperatively via QS. Through the density‐dependent accumulation of small diffusible molecules, microbes sense the size of the local population, and once the surrounding level of quorum‐sensing molecules (QSMs) reach a threshold concentration, a concerted change in gene expression occurs (Waters and Bassler, 2005). This can lead to a switch in the mode of growth, for example, a morphological switch, or biofilm formation, and the expression of virulence factors (De Sordi and Mühlschlegel, 2009). QS has the potential to be pathogenic by two means: firstly, through controlling the population‐wide expression of virulence factors, or secondly, in some instances, through QSMs themselves being directly toxic to host cells (Albuquerque and Casadevall, 2012). Because of their essential involvement in virulence, QS mechanisms are now being targeted for non‐lethal antimicrobial therapies (Raina *et al*., 2009).

Quorum sensing was originally thought to be specific to bacteria, but the investigation of the cell density‐dependent morphological switch in *C*. *albicans* led to the discovery that farnesol acts as QSM in this eukaryote (Hornby *et al*., 2001). Since the discovery of farnesol, QSMs have been described in a number of other fungal species (Hogan, 2006; Tseng and Fink, 2008; Albuquerque and Casadevall, 2012), which are involved in regulating growth, stress resistance, morphogenesis and biofilm formation (Table 1). So far, identified fungal QSMs include peptides, for example, quorum‐sensing‐like peptide 1 of *Cryptococcus neoformans* (Lee *et al*., 2007), oxylipins in *Aspergillus nidulans* (Affeldt *et al*., 2012), and alcohols and alcohol derivatives, such as tyrosol, a phenolic compound that induces filamentation of *C*. *albicans* (Chen *et al*., 2004). Although fungi have not been shown to produce analogues of the bacterial autoinducers (De Sordi and Mühlschlegel, 2009), research into fungal QS is still in its infancy, and it is likely that there are many QS systems still to be discovered.

**Table 1:** Known effects of quorum‐sensing molecules on fungi.

Class	Quorum‐sensing molecule	Known effects	Therapeutic potential	References
Alcohol derivatives	Farnesol	Inhibits morphogenesis and growth	Preventing biofilm formation of bacteria with fungi and augmenting antibiotics	(Hornby *et al*., 2001; Brehm‐Stecher and Johnson, 2003; Derengowski *et al*., 2009; Liu *et al*., 2009; Gomes *et al*., 2011; Brilhante *et al*., 2012; Cordeiro *et al*., 2012; Brasch *et al*., 2013)
		Induces apoptosis	Anti‐tumorgenesis	(Semighini *et al*., 2006; Shirtliff *et al*., 2009; Joo and Jetten, 2010)
		Role in oxidative stress resistance		(Westwater *et al*., 2005)
	Tyrosol	Induces morphogenesis		(Chen *et al*., 2004)
	Dodecanol	Inhibits morphogenesis		(Hogan *et al*., 2004)
		Induces resistance to oxidative stress		(Hall *et al*., 2011)
Acyl‐homoserine lactones	3‐Oxo‐C12 HSL	Inhibits morphogenesis and biofilm formation		(Hogan *et al*., 2004; Mowat *et al*., 2010)
Unsaturated fatty acids	*Burkholderia* DSF	Inhibits morphogenesis	Inhibiting biofilm formation on abiotic surfaces	(Boon *et al*., 2008; Tian *et al*., 2013)
	*Stenotrophomonas* DSF	Inhibits growth		(Kerr, 1996)
Peptides	*Aggregatibacter actinomycetemcomitans* AI‐2	Inhibits morphogenesis		(Bachtiar *et al*., 2014)
	*Streptococcus gordinii* AI‐2	Induces morphogenesis		(Bamford *et al*., 2009)
	*Cryptococcus neoformans* QSP1	Promotes growth and production of virulence factors (e.g. glucuronoxylomannan and melanin)		(Lee *et al*., 2007; Albuquerque *et al*., 2013)

A summary of the current known effects of quorum‐sensing molecules on fungi, including key references.

HSLs, homoserine lactones; DSF, diffusible signal factor; AI, autoinducer; QSP, quorum‐sensing‐like peptide.

### Farnesol

One of the major functions of farnesol is to regulate the morphogenic switch of *C*. *albicans* through modulation of the cAMP‐dependent PKA signalling pathway (Davis‐Hanna *et al*., 2008). Biochemical approaches confirmed that farnesol directly targets the active site of the soluble adenylyl cyclase, inhibiting cAMP production (Hall *et al*., 2011). The significance of farnesol in *C*. *albicans* pathogenicity is still under speculation. One plausible explanation is that farnesol enables yeast cell dissemination from biofilms (Ramage and Saville, 2002). However, the effect of farnesol is not limited to *C*. *albicans*. In fact, farnesol exerts effects on many other fungal species including perturbing the growth of *C*. *neoformans* (Cordeiro *et al*., 2012) and *Penicillium expansum* (Liu *et al*., 2009), inhibiting morphogenesis of *Paracoccidioides brasiliensis* (Derengowski *et al*., 2009) and inducing apoptosis in *A*. *nidulans* (Semighini *et al*., 2006). Furthermore, there is evidence to suggest that farnesol can affect cell wall and cytoskeletal integrity in *A*. *fumigatus* (Dichtl *et al*., 2010). Farnesol and other related alcohols produced by *C*. *albicans* can also inhibit the growth of dermatophytes (Brasch *et al*., 2013), which cause superficial skin and nail infections, including ringworm and athlete's foot (Soll, 2002). Importantly, at high concentrations, farnesol induces apoptosis in *Candida* species (Shirtliff *et al*., 2009), suggesting that farnesol can not only be used to gain a competitive advantage but also as a measure to restrict growth.

In addition to affecting fungal species, farnesol has been shown to mediate effects in bacterial species. For example, farnesol inhibits the production of the *P*. *aeruginosa* quinolone signal (PQS), through inhibition of PqsA (Cugini *et al*., 2007). However, farnesol can also restore PQS production in the absence of LasR, through reactive oxygen species (ROS)‐dependent activation of RhlR signalling (Cugini *et al*., 2010), further complicating the interaction between these two species. The activation of alternative signalling networks in the absence of LasR has been proposed to result from altered bacterial respiration (Cugini *et al*., 2010). Considering that LasR mutants are associated with chronic lung infection in CF patients, the role of these alternative pathways in mediating pathogenicity clearly warrants further investigation.

Because of the wide implications farnesol has on fungal and bacterial growth, it is now being investigated as a potential antimicrobial, including use as an adjuvant alongside antibiotics. For example, farnesol enhances the susceptibility of *S*. *aureus* to various antibiotics (Brehm‐Stecher and Johnson, 2003). In addition, farnesol exhibits synergy with nafcillin and vancomycin to inhibit biofilm formation of *Staphylococcus epidermidis* (Gomes *et al*., 2011; Gomes *et al*., 2011; Pammi *et al*., 2011). Furthermore, farnesol has been shown to augment the efficacy of B‐lactams against *Burkholderia pseudomallei* (Brilhante *et al*., 2012), highlighting not only the potential this QSM has in antimicrobial therapy but also the importance of knowing how these interactions impact on therapeutic treatment.

### N‐Acyl homoserine lactones

*N*‐Acyl homoserine lactones (AHLs, also commonly referred to as homoserine lactones, HSLs) are a QSM produced by Gram‐negative bacteria such as *P*. *aeruginosa*. There are two main proteins involved in AHL‐based QS, LuxI and LuxR; homologues of which also exist in other species (Waters and Bassler, 2005). The AHLs regulate a number of virulence factors within Gram‐negative bacteria, including the expression of competitive antimicrobials, such as phenazines, and the maturation of biofilms (Williams and Cámara, 2009). In addition, they can also significantly alter signalling in eukaryotic cells, as discussed later.

The *P*. *aeruginosa* QSM, 3‐oxo‐C12 homoserine lactone (3‐oxo‐C12 HSL), can inhibit morphogenesis of *C*. *albicans* (Hogan *et al*., 2004) and the conidiation and biofilm formation of *A*. *fumigatus* (Mowat *et al*., 2010). In *C*. *albicans*, *s*imilar to farnesol, 3‐oxo‐C12 HSL mediates its effects through modulation of the fungal cAMP‐dependent PKA signalling pathway (Davis‐Hanna *et al*., 2008). This inhibition of cAMP signalling is due to 3‐oxo‐C12 HSL directly targeting the active site of the soluble adenylyl cyclase, thereby reducing cytoplasmic cAMP concentrations (Hall *et al*., 2011). This interaction is intriguing considering that during direct cell–cell interactions, *P*. *aeruginosa* can bind and kill only *C*. *albicans* hyphae (Hogan and Kolter, 2002), suggesting that the interaction between *C*. *albicans* and *P*. *aeruginosa* is more complex than first thought. One possibility is that *C*. *albicans* evolved this response to avoid being killed. On the other hand, it is possible that the type of interaction that occurs between these microbes is dependent on additional interactions within the environment.

Intriguingly, Peleg *et al*. (2008) observed that *Acinetobacter baumanii*, an emerging multi‐drug‐resistant pathogen found frequently in a nosocomial setting and in the CF lung, inhibited filamentation of *C*. *albicans* within a *Caenorhabditis elegans* infection model. However, deletion of *LuxI* failed to restore yeast morphogenesis, indicating that AHLs produced by *A*. *baumanii* do not modulate hyphal formation in this model. Therefore, it is possible that *A*. *baumanii* produces an alternative and currently unidentified QSM with activity against *C*. *albicans* morphogenesis, or that other factors within the *C*. *elegans* gut interfere with anticipated fungal–bacterial interactions.

In the evolutionary arms race, it appears that some fungi have evolved the ability to inhibit QS. A variety of plant‐mycorrhizal‐associated fungi, from the *Ascomycota* and *Basidiomycota* lineages, can directly interfere with bacterial QS through lactonase‐dependent degradation of QSMs (Uroz and Heinonsalo, 2008). *Trichosporon loubieri* can degrade bacterial AHLs through the production of lactonase (Wong *et al*., 2013). The discovery of lactonase genes in other medically related fungi suggests that AHL degradation may be another dynamic in fungal–bacterial interactions and may have important consequences in colonization and disease, although to our knowledge this area has not been investigated.

### Diffusible signal factor

In addition to the AHLs, some Gram‐negative bacteria communicate using a group of QSMs named diffusible signal factors (DSFs). DSFs are *cis*‐unsaturated fatty acids (Ryan and Dow, 2011). This novel QS system was first described in plant pathogen *Xanthomonas campestris pv*. *campestris* (Xcc) (Barber *et al*., 1997) but has since been identified in human pathogens.

*Cis*‐2‐Dodecenoic acid (or *Burkholderia* diffusible signal factor, BDSF), a QSM produced by the *B*. *cepacia* complex, inhibits the filamentation of *C*. *albicans* (Boon *et al*., 2008; Deng *et al*., 2010). The precise mechanism through which this is achieved has not yet been elucidated. However, Hall *et al*. (2011) showed that BDSF did not act via the same pathway as farnesol or 3‐oxo‐C12 HSL, but worked via the transcriptional repressor Sfl1. Clinical applications based upon this interaction are already being investigated. A recent study indicated that the addition of BDSF could greatly reduce the binding and subsequent biofilm formation of *C*. *albicans* upon abiotic surfaces, including catheters (Tian *et al*., 2013). *Stenotrophomonas maltophilia*, another Gram‐negative bacteria associated within the CF lung also produces a DSF (Valenza *et al*., 2008; Ryan and Dow, 2011; Waters *et al*., 2011). While the interactions of this QSM with fungi has not yet been specifically characterized, it has been found to inhibit the growth of a number of *Candida* species (Kerr, 1996).

**Figure 2:**
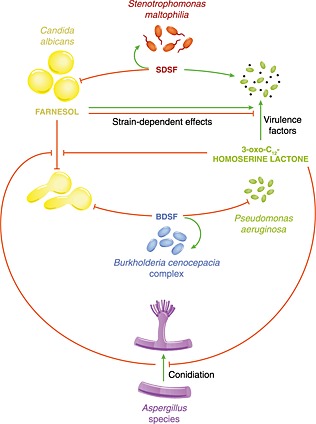
Key interkingdom quorum‐sensing interactions that occur in the cystic fibrosis lung. Diagrammatic representation of quorum‐sensing interactions occurring between fungal and bacterial colonizers of the cystic fibrosis lung. Green lines indicate where a quorum‐sensing molecule exerts a stimulatory effect (i.e. enhanced expression of virulence factors), while red lines indicate inhibition. SDSF, *Stenotrophomonas* diffusible signal factor; BDSF, *Burkholderia* diffusible signal factor, *cis*‐2‐dodecenoic acid.

The QS interactions within the CF lung are complex and dynamic (Fig. 2). As well as the fungal–bacterial interactions already discussed, bacterial QSMs can also interact with each other. For example, the *S*. *maltophilia* DSF can alter the biofilm structure and increase the stress tolerance of *P*. *aeruginosa* (Ryan *et al*., 2008). Furthermore, BDSF can reduce the expression of *P*. *aeruginosa* QS systems and virulence factors, including the type 3 secretion system (Deng *et al*., 2013).

### Autoinducer‐2

Autoinducer‐2 (AI‐2), a family of cyclic oligopeptides, are widely conserved bacterial QSMs with a proposed role in inter‐bacterial communication (Sun *et al*., 2004; Waters and Bassler, 2005). Currently, the role of AI‐2 in fungal–bacterial interactions is confounding. AI‐2 produced by *Aggregatibacter actinomycetemcomitans* inhibits morphogenesis of *C*. *albicans*, similar to BDSF and 3‐oxo‐C12 HSL (Bachtiar *et al*., 2014). However, AI‐2 from *Streptococcus gordinii* promotes morphogenesis of *C*. *albicans* through modulating the effects of farnesol (Bamford *et al*., 2009). These results are intriguing considering that AI‐2 is thought to be structurally conserved among bacteria and is classed as a universal signal (Elias and Banin, 2012). The different responses could be due to study variation (i.e. different media and *C*. *albicans* strain), but a more interesting explanation would be that *C*. *albicans* could distinguish between AI‐2 molecules produced by different bacterial species and amount different responses. The ability of fungi to discriminate between closely related bacterial QSMs has not been investigated. However, given that *C*. *albicans* is a commensal of the gut, where it encounters thousands of bacterial species, the evolution of a system to discriminate between the bacteria would be beneficial to the fungus.

### Enterococcus autoinducers

*Enterococcus faecalis* is a commensal, opportunistic Gram‐positive bacterium that is often found in the same niches as *C*. *albicans*, including the oral cavity and gastrointestinal tract (Cruz *et al*., 2013). Its primary QSM is the gelatinase biosynthesis‐activating cluster peptide, produce via the *fsr* QS system, which is homologous to the well‐characterized Staphylococcal *agr* QS system (Nakayama *et al*., 2006). In a *C*. *elegans* model, the *fsr* system was partially responsible for the inhibition of *C*. *albicans* filamentation, with a number of metabolic genes also playing a role (Cruz *et al*., 2013). Intriguingly, *Enterococcus faecium*, a closely related species, does not inhibit *C*. *albicans* filamentation within the *C*. *elegans* gut (Peleg *et al*., 2008), again suggesting that *C*. *albicans* may have the ability to distinguish between bacterial species.

### Phenazines

Phenazines are secreted toxins. Although not technically QSMs, phenazines are regulated by QS systems and play important roles in fungal–bacterial interactions. For instance, phenazine‐1‐carboxamide produced by *Pseudomonas chlororaphis* has antifungal properties against *Fusarium oxysporum* (Chin‐A‐Woeng *et al*., 1998). In the clinical setting, the four phenazines produced by *P*. *aeruginosa* inhibit the growth of *A*. *fumigatus* through the production of ROS (Briard *et al*., 2015). However, at sub‐inhibitory concentrations, phenazines promote growth of *A*. *fumigatus* via enhanced iron uptake (Briard *et al*., 2015). Phenazine derivatives from *P*. *aeruginosa* are also fungicidal to *C*. *albicans* at high concentrations, but at lower concentrations inhibit fungal morphogenesis and reduce mitochondrial respiration (Morales *et al*., 2013). In fact, phenazine‐1‐carboxamide has been shown to be antifungal against a range of human pathogenic fungi including *C*. *neoformans*, *Candida glabrata* and *A*. *nidulans* (Tupe *et al*., 2015).

## Impact of quorum sensing on the immune system

One important consideration is that the accumulation of these fungal and bacterial QSMs occurs inside the host, and as a result, these QSMs will also affect host cells. For example, farnesol stimulates the NF‐κB pathway via MEK1/2‐ERK1/2‐MSK1‐dependent phosphorylation of p65, leading to production of cytokines including interleukin (IL)‐6 and IL‐1*α* (Joo and Jetten, 2008). In the murine macrophage cell line RAW264.7, farnesol acts synergistically with yeast cell wall components (zymosan) to enhance the expression of proinflammatory cytokines (Ghosh *et al*., 2010). Furthermore, farnesol can alter the maturation of monocytes to dendritic cells (Leonhardt *et al*., 2015). When compared with control treatments, immature dendritic cells cultured in the presence of farnesol were shown to have altered cell surface markers, including increased CD86 and reduced CD1*α*, significantly reduced expression of multiple genes involved in cell adhesion and migration, including *AMICA1* and *MMP2*, and reduced migrational behaviour (Leonhardt *et al*., 2015). These dendritic cells therefore had a reduced capability to recruit and activate T cells, dampening the adaptive immune response. This work highlights the need for a full understanding of the effects of QSMs upon both microbes and their host cells before they could be proposed for therapeutic uses.

Some QSMs can increase stress resistance in fungi, including protecting the fungus from ROS. For example, farnesol has been shown to enhance resistance of *C*. *albicans* to ROS (Westwater *et al*., 2005). This resistance was found to be due to the increased expression of protective catalase Cat1, primarily because of inhibition of the Ras1‐cAMP pathway and cross‐talk with Hog1 regulators (Deveau *et al*., 2010). ROS production is a common mechanism employed by innate immune cells to kill pathogens once inside the phagosome (Flannagan *et al*., 2009). Therefore, it is possible that exposure to QSMs during the course of infection promotes survival of the pathogen inside phagocytes. Farnesol is also able to induce apoptosis in mammalian cells via activation of ROS production (Abe *et al*., 2009). The apoptosis‐inducing effect of farnesol, including the ability to halt cell cycle progression, is being investigated for its anti‐tumorgenesis potential (Joo and Jetten, 2010). Therefore, QSMs may have therapeutic benefits besides controlling microbial growth, and these off‐target effects should be carefully considered before developing QSMs as antimicrobials.

Quorum sensing plays a major role in the immune response during CF (Winstanley and Fothergill, 2009). Similar to farnesol, *Pseudomonas* produced 3‐oxo‐C12 HSL can stimulate the production of cytokines in eukaryotic cells, including IL‐8 in lung fibroblasts and epithelial cells (DiMango *et al*., 1995; Smith *et al*., 2001). IL‐8 is a key cytokine involved in the migration of neutrophils (Huber *et al*., 1991), exacerbating destructive pulmonary inflammation that is a hallmark of CF (LiPuma, 2010).

Within the respiratory tract, mucus and trapped particles are directed away from the lungs by the continued beating on cilia on the surface of epithelial cells, which is dramatically reduced in CF patients. The beating of these cilia is regulated via cAMP (Schmid *et al*., 2007). Considering that both fungal and bacterial QSMs, that are found at high concentrations in CF sputum, have been shown to directly target the activity of adenylyl cyclase (Hall *et al*., 2011), it is tempting to speculate that these QSMs may also influence cilia dynamics, prolonging infection. However, other reports have shown that AHLs can stimulate calcium‐dependent nitric oxide production, increasing mucociliary clearance (Lee *et al*., 2013). These conflicting reports highlight the complexity of the role of QS in host–pathogen interactions.

Another interesting observation is the fact that fungal and bacterial QSMs induce acrosome loss and decrease sperm motility (Rennemeier *et al*., 2009). This suggests that the microbiota of the female reproductive tract may play a role in infertility. However, further studies into the role of the microbiome and QS in infertility are required to confirm these observations.

## Conclusion

In nature, microbes seldom occur in isolation. Instead, microbes grow in communities that may be diverse in species and genera. This interplay has resulted in the evolution of interkingdom polymicrobial interactions. As we delve deeper into the interactions that occur between clinically relevant microorganisms, it becomes clear that these are complex interactions; the effects of which may be dependent on the environment and combination of species present within the niche. So far, research has generally been limited to studying the interactions between dual species (i.e. *C*. *albicans* and *P*. *aeruginosa*). However, in reality, each species is continuously interacting with multiple species at any given time, and currently, we have limited understanding of these dynamics. Because of the already identified cross‐talk between QS systems, it is likely that the outcome of these interactions surpasses the sum of the individual interactions. For example, many of the QSMs that have activity against *C*. *albicans* mediate their effects through modulation of the cAMP‐PKA pathway. Although other signalling cascades have been indicated in fungal QS (Sato *et al*., 2004; Kruppa *et al*., 2004; Kebaara *et al*., 2008), the Ras‐PKA signalling pathway is emerging as a general quorum‐sensing mechanism in fungi. The fungal soluble adenylyl cyclase is responsive to a number of environmental parameters including carbon dioxide (Klengel *et al*., 2005) and bacterial peptidoglycan (Xu *et al*., 2008), which mediate their effects through interactions with different domains of the enzyme. Because of the importance of this signalling pathway in both fungal pathogenesis and interkingdom communication, the fungal soluble adenylyl cyclase has been proposed as a coincidence detector of environmental signals (Hogan and Muhlschlegel, 2011). Further investigation into this area of biology will undoubtedly identify additional QS systems and interactions that play key roles in modulating colonization and disease progression.

It is becoming clear that these microbial communication molecules also exert effects on host cells. The discovery that farnesol enhances proinflammatory responses in macrophages, but prevents activation of cellular immunity, suggests that farnesol is also an immune modulatory signalling molecule. In addition, many pathogens have the ability to replicate within the macrophage phagosome, including *B*. *cenocepacia* (Saini *et al*., 1999), *C*. *glabrata* (Seider *et al*., 2010) and *C*. *neoformans* (Feldmesser *et al*., 2000). Therefore, the phagosome may serve as a micro‐niche enabling QS to occur within mammalian cells. However, our understanding of the role(s) of these QSMs in regulating host–pathogen interactions is still in its infancy. Understanding these interactions, both in terms of their effects on the microbes themselves and on the host, and the role(s) they play in disease is paramount for the development of novel antimicrobial therapies for the treatment of individuals with polymicrobial disease.
